# Aerobic minutes and step number remain low in inpatient stroke rehabilitation

**DOI:** 10.1371/journal.pone.0328930

**Published:** 2025-07-28

**Authors:** Yunyi Yan, Janice J. Eng, Stanley H. Hung, Mark T. Bayley, Krista L. Best, Louise A. Connell, Sarah J. Donkers, Sean P. Dukelow, Victor E. Ezeugwu, Marie-Hélène Milot, Brodie M. Sakakibara, Lisa Sheehy, Hubert Wong, Jennifer Yao, Sue Peters

**Affiliations:** 1 School of Physical Therapy, Faculty of Health Sciences, Western University, London, Ontario, Canada; 2 Rehabilitation Research Program, G.F. Strong Rehabilitation Centre and Department of Physical Therapy, University of British Columbia, Vancouver, British Columbia, Canada; 3 Centre for Aging Smart, Vancouver Coastal Health Institute, Vancouver, British Columbia, Canada; 4 Division of Physical Medicine and Rehabilitation, University of Toronto and KITE Research Institute University Health Network, Toronto, Ontario, Canada; 5 Centre for Interdisciplinary Research in Rehabilitation and Social Integration (Cirris), Centre Intégré Universitaire de Santé et de Services Sociaux de La Capitale-Nationale, Québec City, Quebec, Canada; 6 School of Rehabilitation Sciences, Faculty of Medicine, Université Laval, Québec City, Quebec, Canada; 7 Allied Health Rehabilitation, Lancaster University/East Lancashire Hospitals NHS Trust, Lancashire, United Kingdom; 8 School of Rehabilitation Science, College of Medicine, University of Saskatchewan, Saskatoon, Saskatchewan, Canada; 9 Department of Clinical Neurosciences, University of Calgary, Calgary, Alberta, Canada; 10 Department of Physical Therapy, University of Alberta and Glenrose Rehabilitation Hospital, Edmonton, Alberta, Canada; 11 School of Rehabilitation, Faculté de médecine et des sciences de la santé, Université de Sherbrooke and Research Center on Aging, Sherbrooke, Quebec, Canada; 12 Department of Occupational Science and Occupational Therapy, Vancouver, BC and Centre for Chronic Disease Prevention and Management, Vancouver, British Columbia, Canada; 13 Bruyère Health Research Institute, Ottawa, Canada; 14 School of Population and Public Health, University of British Columbia and Centre for Advancing Health Outcomes, St. Paul’s Hospital, Vancouver, British Columbia, Canada; 15 Division of Physical Medicine and Rehabilitation, University of British Columbia and G.F. Strong Rehabilitation Centre, Vancouver, British Columbia, Canada; 16 Gray Centre for Mobility and Activity, Parkwood Institute, London, Ontario, Canada; Anglia Ruskin University UK, UNITED KINGDOM OF GREAT BRITAIN AND NORTHERN IRELAND

## Abstract

**Objective:**

Rehabilitation is important for regaining mobility poststroke. Clinical practice guidelines suggest a high number of repetitive stepping activities to optimize subacute recovery especially when undertaken at intensities that challenge cardiovascular fitness. However, adherence to these guidelines is unclear. The objective of this study was to quantify aerobic minutes and step number in usual care inpatient stroke rehabilitation unit physical therapy sessions across Canada and identify characteristics of participants who met guideline aerobic intensity minutes at a session midpoint in their rehabilitation.

**Methods:**

To gain insight into usual care, we analyzed cross-sectional data from the usual care arm of the Walk ‘n Watch implementation trial; trial sites included Canadian rehabilitation units that were not typically involved in research studies. To be included, medically stable patients were admitted for inpatient stroke rehabilitation, and able to take > 5 steps with a maximum of one person assisting. We assessed a midpoint physical therapy session with a wrist-based heart monitor (aerobic minutes) and ankle-based step counter (step number). Means, histograms, and correlations between aerobic minutes (> 40% heart rate reserve) and steps were calculated.

**Results:**

There were 166 participants (69 females, age 69 standard deviation (SD)12 years) with stroke (138 Ischemic/ 27 Hemorrhagic) included. Participants had a mean of 10(SD11) aerobic minutes and 985(SD579) steps. The relationship between step number and aerobic minutes was negligible (R^2^ = 0.003). More participants with ≥20 aerobic minutes in a session were male, with lower 6 Minute Walk Test distance, and have a subcortical stroke location.

**Conclusion:**

The number of steps has increased, but aerobic minutes has not changed and remains extremely low compared to published reports in the past several years. Given that increasing activity levels are critical for stroke recovery, further investigation into the potential barriers to achieving targets set by guidelines is recommended.

**Trial registration:**

ClinicalTrials.gov NCT04238260

## Introduction

Stroke is one of the leading causes of disability [1]; each year, there are 13.7 million new cases worldwide [2]. After a stroke, patients may experience deficits in motor function, which can contribute to low levels of walking activity, along with other post-stroke impairments like cognitive and sensory changes. Individuals with motor impairments may experience hemiparesis and declines in balance and coordination that may impact walking and compromise quality of life [1,3]. Thus, regaining walking independence becomes one of the common rehabilitation goals of people post-stroke [4]. Yet, walking activity levels on inpatient stroke units during the early subacute phase of recovery (defined as 7 days to 3 months poststroke [5]) is historically reported as being very low, with the average number of steps per session at 357 (95% CI = 296–418) in 2009 [6], and 63 (range 3–319) in 2012 [7]. It is possible that clinical practice has improved given the many trials, meta-analyses, and guidelines over the last decade that suggest that structured, progressive exercise as safe and effective for improving walking outcomes after stroke [8–10]. For example, in the Determining Optimal post-Stroke Exercise (DOSE) trial [11], we showed that people with stroke can increase their activity levels each week with a structured, progressive protocol [12]. This meant that patients with very slow gait speeds (i.e., 0.2m/s) were able to take >1300 steps in their 10^th^ physical therapy session (approximately the midpoint of a typical 4-week inpatient stroke rehabilitation unit stay in Canada) whereas a patient with mild impairment (i.e., gait speed of 0.6 m/s) reached over 2500 steps, on average [12]. Further, on average patients achieved 27 minutes in at least 40% heart rate reserve (HRR).

Beyond positive impacts on the heart, muscle, and bone, an acute bout of activity at an aerobic intensity generates a transient increase in the level of brain-derived neurotrophic factor, cerebral blood flow, and cortical excitability [13,14]. Importantly, after prolonged periods of aerobic activity, these transient changes in neurotrophic factors, brain blood flow, and excitability can translate into sustained functional improvements in motor control, cardiovascular function, and walking endurance [15,16]. The Stenting and Aggressive Medical Management for Preventing Recurrent Stroke in Intracranial Stenosis (SAMMPRIS) trial showed a dose-dependent effect of exercise after stroke, and specifically, that greater levels of physical activity decreased the likelihood of recurrent stroke, as well as myocardial infarction and vascular death (OR 0.6, 95%CI 0.4–0.8) [17].

A recent clinical practice guideline for people with chronic stroke indicates strong evidence that walking training at moderate to high intensities improves walking function [18]. A meta-analysis also shows that for people with subacute to chronic stroke, moderate to high intensity walking training improves walking speed and endurance [19]. Consequently, patients with a stroke are recommended to exercise during the subacute phase of recovery [5] at 40–70% HRR, three to five times a week for 20–60 minutes per session for optimal stroke recovery [8,9].

Given the wealth of evidence of positive impacts of step number and aerobic intensity on walking recovery and minimal documented safety concerns, it would be important that an adequate number of steps at an aerobic intensity within these recommendations, specifically 40–70% HRR 3-5x/week for 20–60 minutes per session [8,9], be delivered during usual care clinical practice. However, the relationship between steps and aerobic minutes for people in the subacute phase of recovery after stroke is unclear. In people with chronic stroke, higher stepping intensities (defined as ≥30 steps per minute) are linked to aerobic outcomes, specifically cardiovascular fitness [20]; however, this study did not specifically examine this relationship in people with subacute stroke.

Thus, the primary aim of our study was to quantify the time in minutes of aerobic exercise (>40%HRR) and number of steps taken during a usual care midpoint physical therapy session across multiple inpatient stroke units in Canada and determine whether there was a relationship between these variables. Usual care in Canada consists of 5 day/week of 30–60 minutes of physical therapy for 3–4 weeks, so a midpoint physical therapy session was chosen (~10^th^ session). Second, we aimed to describe the characteristics of those who achieved the recommended minimum duration of aerobic intensity (i.e.,* *≥ 20 minutes) at this session.

## Methods

### Study design and population

This cross-sectional study is a secondary data analysis of 12 Canadian sites involved in a clinical trial called Walk ‘n Watch (trial registry at www.ClinicalTrials.gov ID: NCT04238260). Data will be made available in the Borealis Canadian Dataverse Repository (https://borealisdata.ca/dataverse/eng). Walk ‘n Watch is a stepped wedge clinical trial where each site collected outcomes in Usual Care, and then, switched to the Walk ‘n Watch protocol which focuses on completing a minimum of 30-minutes of weight-bearing, walking-related activities (at the physical therapists’ discretion) that progressively increases in intensity informed by activity trackers measuring heart rate and step number [21]. All of the data from the Usual Care arm are presented here. Canadian rehabilitation sites outside of regions typically involved in research studies were included to gain insight into usual care. Usual care in Canada consists of 5 day/week of 30–60 minutes of physical therapy. Complete details of the research protocol for the Walk ‘n Watch trial have been previously published [21]. Local research ethics boards from the following sites approved the research protocol and written consent was obtained from each participant in accordance with the Declaration of Helsinki.

- Harmonized ethics from the Clinical Research Ethics Board (CREB) at the University of British Columbia (H19-02809), Vancouver, British Columbia, Canada;
- Bruyère Research Ethics Board at the Bruyère Health – Élisabeth Bruyère Hospital (M16-20-015), Ottawa, Ontario, Canada;
- Research Ethics Board at Centre integre universitaire de sante et de services sociaux (MP-13-2020-1947), Québec, Québec, Canada;
- Research Ethics Board at Centre integre universitaire de sante et de services sociaux de l’Estrie – Centre hospitalier universitaire de Sherbrooke (MEO-13-2022-458), Sherbrooke, Quebec, Canada;
- Horizon Health Network Research Ethics Board at Dr. Everett Chalmers Regional Hospital (ROMEO File #: 100731), Fredericton, New Brunswick, Canada;
- Waterloo-Wellington Research Ethics Board (WWREB) at Grand River Hospital-Freeport Campus (WWREB Study #: 2021-0741), Kitchener, Ontario, Canada;
- The Health Research Ethics Board (HREB) at University of Alberta (Pro00097418), Edmonton, Alberta, Canada;
- Joseph Brant Hospital Research Ethics Committee at Joseph Brant Hospital (ID #: 000-053-20), Burlington, Ontario;
- PEI Research Ethics Board at Queen Elizabeth Hospital, Charlottetown, PEI, Canada;
- Biomedical Research Ethics Board (Bio-REB) at University of Saskatchewan (Application ID: 1673), Saskatoon, Saskatchewan, Canada

Broad inclusion criteria were implemented to capture the realistic caseload of stroke rehabilitation units for patients who had walking as a rehabilitation goal. In this study, patients recruited to the study were medically stable (e.g., no active cancer, stable cardiovascular condition), within 12-weeks from their date of stroke, able to walk a minimum of 5 steps with up to maximal assistance from one therapist, and able to understand English and/or French. Patients with another neurological disorder (e.g., Parkinson’s disease) or registered in another rehabilitation study were excluded.

### Outcomes

At baseline, demographic information, including sex, age, and resting heart rate, type and location of stroke, and walking endurance (6-Minute Walk Test, 6MWT) was collected. The typical length of stay on an inpatient rehabilitation unit in Canada is 3–4 weeks [22] with 5 days/week of physical therapy; therefore, a midpoint physical therapy session two weeks after enrollment in the study was selected (~10^th^ session). To measure step counts in this physical therapy session, each participant wore a StepWatch™ (Modus Health, USA) on their non-paretic ankle. The StepWatch™ has high validity when placed on a participant’s non-paretic ankle [23], and high reliability with manual step counts in healthy adults (ICC 0.99) [24]. Also, participants wore a Garmin Forerunner 235 watch (Garmin Ltd., USA) on their non-paretic wrist to measure their aerobic minutes defined as the number of minutes at >40% HRR [9]. Our definition of aerobic minutes, > 40% HRR, was based on the recommendations outlined in Billinger et al 2014 and MacKay-Lyons et al 2020 [8,9]. In healthy adults, the Garmin Forerunner 235 has high agreement with electrocardiogram (Lin’s concordance correlation coefficient, rc = 0.81) [25].

### Physical therapy

Each participant received standard physical therapy (Usual Care) at the discretion of their physical therapists. Before the session, a research coordinator at each site programmed the Garmin watch with participant-specific age-predicted maximal heart rate and measured resting heart rate to individualize their HRR recording to >40%HRR, and positioned the Garmin watch as well as the StepWatch™ on the participant’s non-paretic wrist and ankle, respectively. Age-predicted maximal heart rate used the following formulas as outlined in MacKay-Lyons et al 2020: [9] 1) without beta-blockers: 206.9 – (0.67 x age), and 2) with beta-blockers: 164 – (0.7 x age). The step counter and heart rate monitor were worn for the entire session.

### Statistics

Descriptive statistics were used to characterize the sample. Data were categorized into histograms denoting the number of participants who achieved aerobic minute (0–5 min, 6–10 min, 11–20 min, > 20min), and step number categories (0–500steps, 501–1000steps, 1001–1500steps, 1501–2000steps, > 2000steps). Histograms were used to display the shape of the distribution of the data, and the spread of values. A scatter plot that compared step number and aerobic minutes was created and a coefficient of determination (R2) was calculated. Descriptive statistics, plots, and coefficient of determination (R2) were conducted in Microsoft Excel (v. 16.76).

## Results

Recruitment for the primary outcome of the randomized controlled trial has been completed. Across 7 provinces and 12 sites, a total of n = 1442 patients were screened with n = 284 determined to be eligible, and n = 167 enrolled in the study into usual care between 01/06/2021 and 01/01/2023. One participant withdrew before baseline testing; thus, a total of 166 participants were included in this study. See Table 1.

**Table 1:** Demographics and clinical characteristics of sample.

Demographics	n = 166
**Sex (F/M)**	69F/ 96M/1 missing data
**Mean (SD) age, years**	69 (12)
**Stroke type**	138 Ischemic/ 27 Hemorrhagic/ 1 participant with missing data
**Mean (SD) resting heart rate, bpm**	73 (12)
**Mean (SD) heart rate, bpm**	92 (15)
**Mean 6MWT (m, SD), range**	140(101) range 0–448
**Mean (SD) aerobic minutes**	10 (11) 24 participants with missing data
**Mean (SD) step number**	985 (579) 23 participants with missing data
**Mean (SD) Stroke to Inpatient Rehabilitation Admission, days**	18 (13)
**Mean (SD) Inpatient Rehabilitation Hospital Length of Stay, days**	42 (24) 1 participant with missing data

Legend: bpm = beats per minute, F = female, M = male, SD = standard deviation, m = metres.

We did have several participants with missing data for steps or aerobic minutes. Of the missing sessions, three patients had medical issues (e.g., going into quarantine because of a COVID-19 diagnosis), five patients were discharged earlier than anticipated before a typical session could be measured, and one patient dropped out of the study. About 10% of the sessions were missing due to technical difficulties with using the devices (3 for the StepWatch™, 7 for the Garmin watch) and there were some sessions without reasons provided for the missing data (11 for steps, 9 for aerobic minutes).

**Fig 1:**
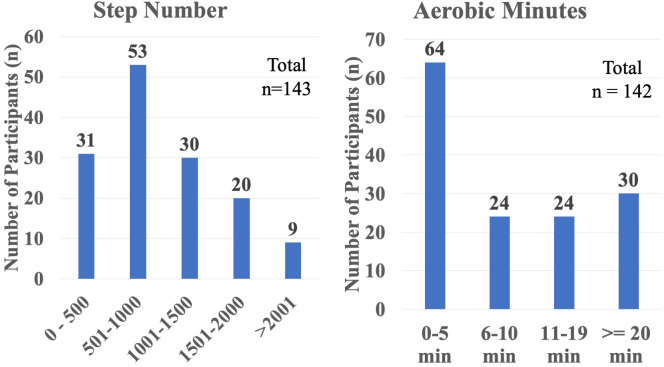
Histogram of the number of participants within each category of step number (left) and aerobic minutes (right).

**Fig 2:**
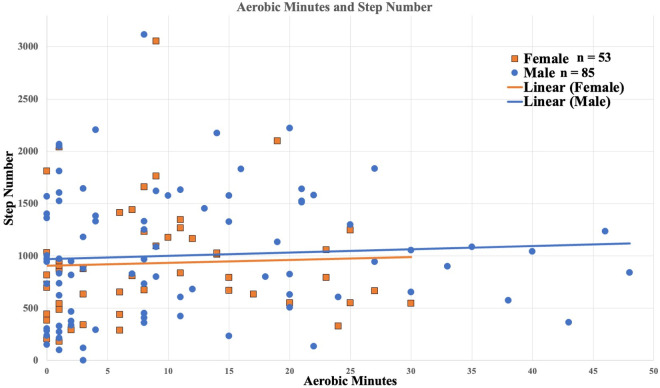
Relationship between step number and aerobic minutes. Female data points are orange squares with male data points in blue circles. Linear trend lines are in orange for females and blue for males.

Participants achieved an average of 985 steps (95% CI 897–1073), with a median of 872 steps. For aerobic minutes, participants had an average of 10 minutes (95% CI 8–12), with a median of 8 minutes (mid quartile range of 1–15 minutes) (Fig 1). The relationship between step number and aerobic minutes was negligible (R^2^ = 0.003) (Fig 2).

The characteristics of participants with greater than or equal to 20 aerobic minutes per session compared to those with less than 20 minutes are in Table 2. There were 30 participants with ≥20 minutes (18% of the sample). Descriptively, more males than females, and more participants who had lower baseline walking endurance (6MWT distance), achieved the recommended aerobic minutes. For stroke characteristics, there were more participants with ≥20 minutes with subcortical stroke locations than cortical. Other demographics were similar between these groups.

**Table 2:** Characteristics of participants with ≥20 minutes in a midpoint session compared to those with less than 20 minutes.

	≥20 minutes (n = 30)	<20 minutes (n = 136)
Sex: M,F (count, %)	22, 8 (73%M, 27%F)	74, 60 (55%M, 45%F)
Age (mean, SD)	70(11)	68(13)
Baseline 6MWT (meters, SD)	114(85)	146(110)
Resting HR (mean, SD)	74(13)	73(12)
Stroke Type (count, %)	24 I, 6 H (80%I, 20%H)	113 I, 21 H (84% I, 16% H)
Stroke Location (count, %)	5 C, 22 SC (19% C, 81% SC)	39 C, 63 SC (38% C, 62% SC)
Aerobic Minutes (mean, SD)	28(8)	5(5)
Steps (mean, SD)	945(479)	992(597)

Legend: 6MWT = 6-Minute Walk Test, C = cortical, F = female, H = hemorrhagic, I = ischemic, M = male, mRS = modified Rankin Scale, SC = subcortical, SD = standard deviation.

## Discussion

Our primary aim was to quantify the step number and aerobic minutes in a midpoint inpatient physical therapy session, determine if a relationship between these variables was present, and describe the characteristics of those participants who met the daily aerobic minute guidelines of ≥20 minutes within a single session. We found that most participants had 10 or less aerobic minutes with less than 1000 steps in one physical therapy session, and no relationship between the number of steps and aerobic minutes. For the 18% of our participants who did achieve ≥20 aerobic minutes, there were more participants who were males with subcortical stroke locations and have lower baseline walking endurance. Considering the recommendation of a minimum of 20 aerobic minutes per session a minimum of three days weekly published in 2014 [8], and updated in 2020 [9], the majority of our participants achieved much less with approximately 44.8% of our participants with fewer than five aerobic minutes.

Overall, step counts have changed over time. When we compared our data to published reports conducted in the subacute recovery phase after stroke, we observed an increase in step number per session [13] and our results are within the range of more recently published studies [11]. Studies fifteen years ago indicate between 63 and 357 steps/session, increasing to 168–1167 in the last 3 years. This represents a 3-fold increase in step number. The average step count in our study physical therapy sessions (985 steps) were lower than the average step counts (1167 steps) reported in 2020 by Moore and colleagues [26] but higher than the average of 580 steps reported in 2020 by Klassen and colleagues [11]. The average step counts in the current study are larger than the median step count of 63 steps near discharge reported in 2012 [7], indicating an increase in inpatient activity levels over time. It is possible that efforts to disseminate knowledge of current activity guidelines are beginning to take effect.

The physical activity levels for aerobic minutes in inpatient physical therapy during the subacute recovery period have not changed in the last few years. Twenty years ago, 3 aerobic minutes were reported by MacKay-Lyons and Makrides [13] which has increased to 11 minutes in the last 3 years [11]. The average aerobic minutes in our participants was 10 minutes suggesting little change, and remains below the recommendation of a minimum of 20 minutes. Overall, the absolute increase in aerobic minutes from twenty years ago to now is very low and does not represent a meaningful increase in aerobic activity within physical therapy sessions.

There may be several individual- and system-level contributing factors to the low number of steps and aerobic minutes observed. For individual factors, physical therapists may target multiple functional goals within one session, and may have prioritized other therapy goals (i.e., upper limb recovery, discharge planning) and activities based on the patient’s presentation. Increasing step counts and heart rate intensities often conflict with therapists’ beliefs on the need for therapy time spent on pre-gait activities and ensuring movement quality versus quantity [27,28]. Therapists may have also had safety concerns with aerobic exertion for those with cognitive or physical impairments and cardiac disease [28,29]. For system-level barriers, therapists have reported feeling more comfortable with aerobically exerting patients after screening with formal graded exercise stress testing and access to equipment to monitor heart rate [28]. However, resource limitations often preclude the possibility of formal stress testing and access to heart rate monitors in inpatient rehabilitation units [30]. For example, these stress tests are often not available or feasible, particularly in more remote/rural hospitals. Therapists have also reported that higher aerobic minutes and step counts may be more feasible with more time and clinical staffing [28]. In some settings, other members of the health care team, such as therapy assistants, may be involved with delivering aerobic activity. Taken together, there are probably multiple reasons why the number of steps and aerobic minutes are low. Further research to understand the barriers to increasing physical activity levels is warranted, as well as ways to support delivering increases in steps and aerobic minutes. Indeed, several research groups are conducting research in this area [27,28].

While these are substantial gains in step activity, these current activity levels were still below the recommended guidelines of 20–60 aerobic minutes at 40–70% HRR for the subacute recovery phase [8,9]. Physical activity in inpatient rehabilitation during the subacute recovery phase after stroke is essential to drive recovery; however, we found it is currently inadequate for most participants to meet guideline recommendations. This may mean that it poses a challenge for patients to remain physically active after discharge.

Demographically, there were more males with subcortical stroke locations with lower baseline walking endurance for the 18% of our participants who did achieve ≥20 aerobic minutes (Table 2). It is possible that those participants with lower 6MWT distance at baseline had higher aerobic minutes because it may be more aerobically demanding to achieve a similar number of steps compared with participants with higher 6MWT distances. Also, it is possible that higher functioning participants had therapy time devoted to other activities (e.g., the upper limb, activities of daily living). There were more males than females who achieved ≥20 aerobic minutes (22 males vs 8 females). The reasons for this are likely complex and outside of the scope of this study. However, based on the literature, females tend to have worse functional outcomes at rehabilitation discharge including worse standing balance, slower gait speed, and higher fatigue levels [31–33], all of which may impact the number of aerobic minutes within a therapy session. More participants with subcortical lesions versus cortical lesions were able to obtain ≥20 aerobic minutes. It is unclear how this may impact the number of aerobic minutes specifically, so future studies may wish to examine this more directly. Though there were more participants who were male, had lower baseline walking endurance, and subcortical stroke locations, these results should be interpreted with caution as it represents only 18% of our total sample (30 participants vs. 136 participants, Table 2). Future research could be undertaken to understand these potential demographic differences in who achieves the recommended aerobic minutes.

There are a few limitations that should be acknowledged with this study. Since we measured one session, our results only provided a snapshot of the activity levels within current inpatient physical therapy practice at the clinical sites included in the Walk ‘n Watch trial. It is possible that participants had higher step numbers or aerobic minutes in other physical therapy sessions or in sessions with physical therapy assistants, or across a full day. The accuracy of our measurement tools may also be a potential limitation. However, the StepWatch™ has high criterion validity compared with lab-based 3D gait analysis in people with stroke [23], and high accuracy compared with manual step counts [24]. In young healthy adults, for heart rate the Garmin Forerunner had high correlation with electrocardiogram (ECG) across various aerobic exercise conditions (Lin’s concordance correlation coefficient, rc = 0.81) [25]. However, to our knowledge, the relationships between the Garmin and ECG in people with stroke has not been established so it is possible that aerobic minutes are under or over reported here. Further, some sessions had missing data which may have impacted the findings. It is also possible that the use of certain medications, like beta blockers, may have affected activity levels during the session. Therapists were not masked to the participants being monitored, which may mean that they changed their typical approach to activities within the session. Lastly, there is variability amongst physical therapists in how therapy sessions are operationalized. Future studies should collect data on the characteristics of the therapists who implemented the sessions to better understand this element of activity levels.

## Conclusions

Most participants in our study did not meet current guidelines with less than 10 aerobic minutes and 1000 steps in one physical therapy session; yet, overall, the number of steps in a session of physical therapy appears to have increased in the past several years. Given that increasing activity levels are critical for recovery post stroke, further investigation into the potential barriers to achieving steps and aerobic activity intensity targets along with methods to implement practice changes are recommended.

## Supporting information

S1 Checklist This supporting information is the Human Participants Research Checklist. (DOCX)
